# Draft whole-genome sequences of ancestral and modern *Mycobacterium tuberculosis* Beijing strains with different patterns of virulence

**DOI:** 10.1128/mra.00211-25

**Published:** 2025-12-12

**Authors:** Lia Gomes, Edson Machado, Michel Abanto Marin, Sidra Vasconcellos, Antonio Basílio de Miranda, Igor Mokrousov, Elena Lasunskaia, Philip Suffys

**Affiliations:** 1 Laboratory of Molecular Biology Applied to Mycobacteria, Oswaldo Cruz Institute, Oswaldo Cruz Foundation 196605 , Rio de Janeiro, Brazil; 2 Genomics and Bioinformatics Unit, Scientific and Technological Bioresource Nucleus (BIOREN), Universidad de La Frontera 28057, Temuco, Chile; 3 Pole Computational Biology and Systems, Oswaldo Cruz Institute, Oswaldo Cruz Foundation 196605 , Rio de Janeiro, Brazil; 4 Laboratory of Molecular Epidemiology and Evolutionary Genetics, St. Petersburg Pasteur Institute 349158, Saint Petersburg, Russia; 5 Laboratory of Biology of Recognition, Universidade Estadual do Norte Fluminense 28109, Campos, Rio de Janeiro, Brazil; University of Maryland School of Medicine, Baltimore, Maryland, USA

**Keywords:** *Mycobacterium tuberculosis*, Beijing, genome, virulence

## Abstract

*Mycobacterium tuberculosis* of the Beijing Modern Sublineage displays increased virulence when compared with strains of the Ancient Sublineage. Here, we report the full genome of strains of the two sublineages isolated from tuberculosis patients from Brazil, Mozambique, and Russia.

## ANNOUNCEMENT

The Beijing subtype of *Mycobacterium tuberculosis* (*Mtb*B) lineage 2 is causing tuberculosis (TB) in patients residing in large part of the world and has been associated with outbreaks and drug-resistant TB (1). Tuberculosis caused by *Mtb*B strains has the highest prevalence in East and South Asia, South Africa, and countries of the former Soviet Union (1, 2). In Latin America, about 3.9% of the patients are infected with *MtbB,* with the highest incidence in Cuba (17%), Peru (16%), and Colombia (5%); Brazil has a rate lower than 1% (3). Modern and ancient strains of *Mtb*B are differentiated on a molecular level and show distinct evolutionary and phylogenetic characteristics (4–6).

Here, we present the draft genome sequences of eight *Mtb*B strains isolated between 2002 and 2009. Three strains from native TB patients attending primary health care clinics in Rio de Janeiro and Sao Paulo in Brazil (5) Four isolates were from patients in the Maputo province in Mozambique (7). One modern *Mtb*B strain was from a patient with TB in Russia provided by St. Petersburg Research Institute of Phthisiopulmonology (5).

The isolates were obtained from sputum samples and grown in Lowenstein-Jensen medium at 37°C for 28 days and characterized as being *Mtb*B by spoligotyping. To differentiate ancient and modern sublineages, we evaluated the presence and number of IS6110 copies in the NTF region (Table 1) (8, 9).

**TABLE 1:** Genomic characteristics of the *Mtb*B strains included in this study^*a*^

Strain	Family	Geographic origin	Sublineage	Insertion of IS6110 in NTF region	Number of reads (R1 + R2)	Reads after trimming (R1 + R2)	GC	Genome size	Coverage	N50	L50	ORFS	Completeness	Bioproject	Accession	Illumina SRA	GenBank accession
1471RU	Beijing	Russia	Modern	One insertion	3,003,452	1,985,708	0.655632	4,402,857	45.01	45,826	31	4495	99.86	PRJNA310858	SAMN04456659	SRR34103930	LWSS00000000
3912BR	Beijing	Brazil-RJ	Ancient	No insertion	15,446,472	7,890,336	0.655311	4,329,376	178.85	57,934	25	4332	99.94	PRJNA310866	SAMN04456708	SRR34103314	LUKV00000000
2172BR	Beijing	Brazil-RJ	Modern	One insertion	18,449,924	10,758,360	0.655392	4,333,702	243.86	53,712	27	4323	99.94	PRJNA310865	SAMN04456663	SRR34103313	LTZI00000000
ZT264BR	Beijing	Brazil-SP	Ancient	No insertion	4,001,018	1,009,406	0.651721	4,345,914	22.88	22,659	62	4534	99.47	PRJNA296585	SAMN04100772	SRR34097737	JBJYTZ000000000
442MO	Beijing	Mozambique	Ancient	No insertion	10,843,390	5,311,694	0.655463	4,336,351	120.4	67,716	18	4305	99.94	PRJNA310867	SAMN04456709	SRR34103315	LTZH00000000
16MO	Beijing	Mozambique	Modern	One insertion	5,724,914	4,366,652	0.655836	4,342,984	98.98	98,868	16	4259	99.94	PRJNA310877	SAMN04456795	SRR34097436	LUKS00000000
198MO	Beijing	Mozambique	Modern	One insertion	28,336,220	9,290,234	0.649585	4,457,680	210.58	50,315	28	4447	99.94	PRJNA310875	SAMN04456734	SRR34097437	VHOB00000000
284MO	Beijing	Mozambique	Modern	One insertion	21,805,210	10,203,462	0.655487	4,333,902	231.29	61,134	24	4324	99.94	PRJNA310870	SAMN04456719	SRR34097435	LUKT00000000

^*a*^ The average sequencing coverage was calculated as (A × B) / Ref, where A is the number of reads after trimming, B is the average read size (100 bp), and Ref is the genome size of *Mycobacterium tuberculosis *H37Rv (4,411,532 bp; GenBank accession NC_000962.3).

For genomic sequencing, two bacterial loops were obtained from the Lowenstein-Jensen culture under the same conditions as before and DNA was extracted using the Charge Switch gDNA bacteria DNA Mini Kit (Invitrogen), with an elution process without EDTA, which can inhibit enzymes, leading to low fragmentation and failure in library preparation (Thermo Scientific). DNA quality control was performed by Nanodrop quantification to detect possible protein contamination, and for precise quantification of 1 ng/µL, a Qubit (Thermo Scientific) was used. Paired-end libraries were prepared using the Nextera XT DNA library kit after DNA was fragmented and ligated to adapters by enzymes, generating fragments of around 100 bp. Sequencing was performed on an Illumina HiSeq 2500 at the High Throughput Sequencing Platform of the Oswaldo Cruz Institute (Fiocruz, RJ).

For further analysis, default parameters were used for all software unless otherwise specified. Initial quality control of raw reads was performed using Trimmomatic v0.39 (10), with the following parameters: LEADING:3, TRAILING:3, SLIDINGWINDOW:4:20 and MINLEN:40. Before and after trimming, the reads quality was verified using FastQC v0.12.1 (11), and genomes were *de novo* assembled with SPAdes v3.515 (12). We used CheckM v1.2.2 (13) to check the quality and completeness of genomes, and the annotation was performed by the NCBI Prokaryotic Genome Annotation Pipeline (PGAP) v6.9 (14).

Experimental models show that modern strains are more virulent, with more pathogenicity factors, higher bacterial loads, and greater mortality than ancestral strains (5, 9). In a previous study, we published the genome comparison of *Mtb*B isolates from two patients from Brazil, one being of the ancient and the other with the modern genotype and a greater number of SNPs in genes associated with virulence being observed in the modern strain (15). We now present the draft genomes of part of these strains in the hope that this might contribute to better-defined *Mtb*B-associated virulence factors.

## Data Availability

NCBI accession numbers are provided in Table 1.
